# A Double-Blind Randomized Clinical Trial on the Efficacy of Cortical Direct Current Stimulation for the Treatment of Alzheimer’s Disease

**DOI:** 10.3389/fnagi.2014.00275

**Published:** 2014-10-09

**Authors:** Eman M. Khedr, Nageh F. El Gamal, Noha Abo El-Fetoh, Hosam Khalifa, Elham M. Ahmed, Anwer M. Ali, Mostafa Noaman, Ahmed Abd El-Baki, Ahmed A. Karim

**Affiliations:** ^1^Department of Neurology, Assiut University Hospital, Assiut, Egypt; ^2^Department of Prevention and Health Psychology, University of Riedlingen, Riedlingen, Germany; ^3^Department of Psychiatry and Psychotherapy, University Clinic Tübingen, Tübingen, Germany

**Keywords:** Alzheimer’s disease, cognitive function, transcranial direct current stimulation, Wechsler Adult Intelligent Scale, cortical excitability, auditory event-related potentials (P300), cortical plasticity

## Abstract

**Background:** The purpose of this study was to investigate the long-term efficacy of transcranial direct current stimulation (tDCS) in the neurorehabilitation of Alzheimer’s disease (AD).

**Methods:** Thirty-four AD patients were randomly assigned to three groups: anodal, cathodal, and sham tDCS. Stimulation was applied over the left dorsolateral prefrontal cortex for 25 min at 2 mA, daily for 10 days. Each patient was submitted to the following psychometric assessments: mini-mental state examination (MMSE) and Wechsler adult intelligence scale-third edition at base line, at the end of the 10th sessions and then at 1 and 2 months after the end of the sessions. Motor cortical excitability and the P300 event-related potential were assessed at baseline and after the last tDCS session.

**Results:** Significant treatment group × time interactions were observed for the MMSE and performance IQ of the WAIS. *Post hoc* comparisons showed that both anodal and cathodal tDCS (ctDCS) improved MMSE in contrast to sham tDCS. Whereas, this was only true for ctDCS in the performance IQ. Remarkably, tDCS also reduced the P300 latency, but had no effect on motor cortex excitability.

**Conclusion:** Our findings reveal that repeated sessions of tDCS could not only improve cognitive function but also reduce the P300 latency, which is known to be pathologically increased in AD.

## Introduction

Alzheimer’s disease (AD) is a progressive neurodegenerative disorder (Alzheimer’s Association et al., 2011) that causes deficits in many cognitive activities such as memory, language, and executive function including working memory, cognitive flexibility (Monsell, 2003), planning (Chan et al., 2008), and abstract reasoning (Bruce and Jeffrey, 2007). AD’s behavioral effects result from changes in neuronal activity secondary to the disease process itself. There are changes in modulatory transmitter systems, and network connectivity while physiological studies have reported that hyperexcitability of the motor cortex (Di Lazzaro et al., 2004; Rossini et al., 2007; Khedr et al., 2011) correlates with cognitive severity (Alagona et al., 2001).

Given the limited efficacy of pharmacological treatments (Birks, 2006), non-pharmacological approaches in AD are of great interest. One approach that has been used in several centers is repetitive transcranial magnetic stimulation (rTMS), a non-invasive form of brain stimulation that produces after-effects on cortical excitability thought to involve forms of long-term potentiation/depression (LTP/LTD) at central synapses. In most studies, rTMS has been targeted over the dorsolateral prefrontal cortex (DLPFC), given its recognized role in executive function, and has been reported to improve memory, language, and executive functions for several weeks after a period of treatment (Cotelli et al., 2006, 2008, 2011; Ahmed et al., 2012). Rabey et al. (2013) combined TMS (over left and right DLPFC, Broca, and Wernicke areas) with cognitive training (rTMS-COG) on 15 AD patients in a randomized double-blind, controlled study, and observed an improvement in memory and learning enhancement.

However, rTMS is a difficult treatment to sham successfully since real stimulation causes scalp sensations and muscle twitches that are not present with most forms of sham-rTMS. In more recent years, however, a second method has attracted substantial interest, transcranial direct current stimulation (tDCS), which can modulate cortical excitability and induce effects that outlast the period of stimulation which, like those after rTMS, are thought to be due to effects on synaptic long-term potentiation/depression (Bindman et al., 1962; Gartside, 1968; Karim et al., 2003, 2004). tDCS is usually applied using a current of 1–2 mA. Many studies have shown that tDCS at 1 mA often leads to polarity-specific effects, with cathodal tDCS (ctDCS) decreasing and anodal tDCS (atDCS) increasing cortical excitability. However, the duration and strength of tDCS after-effects depend on duration and intensity of the applied currents (Nitsche and Paulus, 2000, 2001; Nitsche et al., 2003; Suemoto et al., 2014; Wiethoff et al., 2014). For example, although 2 mA atDCS over motor cortex seems to produce a slightly longer lasting facilitation of excitability than 1 mA, ctDCS at 2 mA *reverses* the “usual” (1 mA) inhibitory effect to facilitation (Batsikadze et al., 2013). Whether this effect also occurs over other cortical areas is unknown.

Some studies have investigated the effects of (the presumed excitatory) atDCS in AD, in which the left temporal cortex (TC) was targeted because of its role in memory processes, and the DLPFC because of its role in executive function. Boggio et al. (2009) found positive effects on visual recognition memory in 10 AD patients after atDCS at 2 mA for 30 min over the left DLPFC. Another study reported improved word-recognition memory in 10 patients with probable AD based on diagnostic criteria from the National Institute of Neurological and Communicative Disorders and Stroke and the AD and Related Disorders Associations (NINCDS–ADRDA) (McKhann et al., 1984), after atDCS at 1.5 mA for 15 min of the temporoparietal areas (Ferrucci et al., 2008). Finally, enhanced long-term visual recognition memory for up to 4 weeks after therapy was found after atDCS at 2 mA over TC bilaterally in 15 AD patients (Boggio et al., 2012). Furthermore, Cotelli et al. (2014) recorded improvement in naming accuracy after application of atDCS over the left DLPFC with language training in 16 patients suffering from primary progressive aphasia.

We therefore decided to conduct a larger study on 34 patients with AD, to examine the long-term effects of 2 weeks tDCS over the left DLPFC on cognitive function in AD. We chose to apply 2 mA tDCS because many positive clinical studies have used this intensity. However, since both 2 mA atDCS and 2 mA ctDCS are excitatory on the motor cortex (Batsikadze et al., 2013), we decided to compare whether one would be more effective than the other in treating AD. The patients were therefore divided into three groups, which received either atDCS, ctDCS, or sham tDCS applied over the DLPFC daily for 10 sessions. Cognitive function was tested with the Mini-mental state examination (MMSE) (Folstein et al., 1975) and the Wechsler adult intelligence subscales (WAIS-III) (Wechsler, 1997). However, since the neurophysiological mechanisms underlying tDCS modulation of cognitive function is not well understood, we also explored the effects of tDCS on electrophysiological brain activity using the auditory P300 evoked potential, which has been used as an objective biological marker of AD (Parra et al., 2012). Finally, since previous work had shown that AD is associated with increased excitability of motor areas we also examined possible effects on the motor cortex, reasoning that treatment for several days might have wide ranging effects on brain function at a distance from the direct site of stimulation.

## Methods

**Figure 1:**
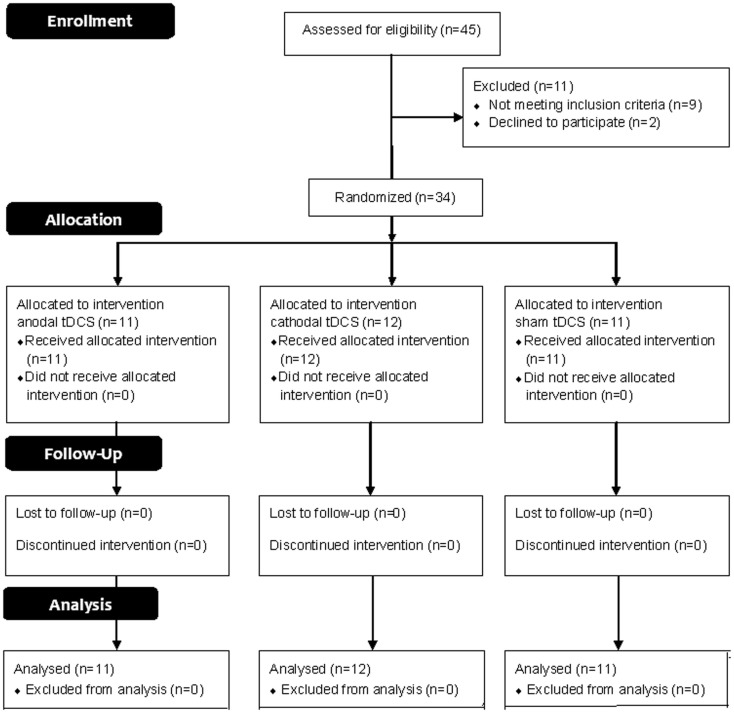
**The flow chart**.

This trial is reported following 2010 CONSORT guidelines. A participants’ flow diagram is shown in Figure 1.

### Patients

Forty-five consecutive patients with a diagnosis of probable AD according to the criteria of the National Institute of Neurological and Communicative Disorders and Stroke Alzheimer Disease and Related Disorders Association (NINCDS-ADRDA) (McKhann et al., 1984), were recruited from out-patients clinics and private clinics during the period from October 2011 to September 2012. In all patients, computed tomography scan (CT) or magnetic resonance imaging (MRI) were obtained to detect the diffuse brain atrophy and to exclude other causes of dementia.

Exclusion criteria were the following: previous history of stroke, metabolic disturbance, other major medical illnesses or epilepsy, and severe forms of dementia. Patients with metallic objects in the body, or subjected to a craniotomy in the past, were also excluded. At the time of recruitment, none of the patients were taking cholinomimetics, antidepressants, or neuroleptic, sedative-hypnotic drugs for at least 1 week before the assessment.

The stage of dementia was evaluated by means of the MMSE (Folstein et al., 1975). Eleven patients were excluded because nine out of them had severe dementia and two patients refused to participate in the study. Thus, 34 patients with mild to moderate dementia were included in this study. Dementia was classified according to the MMSE score as 21–17 for mild, 16–9 for moderate in illiterate patients; these values correspond to the values of 23–19, and 18–11 for mild, and moderate dementia, respectively, using the full score of 30 points in case of educated patients (Farrag et al., 1998).

The mean (SD) age was 69.7 (4.8) years ranging from 62 to 79 years and the mean duration of illness was 3.1 (2.1) years ranging from 1 to 10 years. Nineteen males and fifteen females were including in the study. The mean (SD) total MMSE score of AD patients was 18.1 (3.3) ranging from 12 to 23, 15 patients had mild dementia, 19 patients had moderate dementia.

Each patient was evaluated with the MMSE (Folstein et al., 1975), and WAIS-III (Wechsler, 1997).The WAIS-III provided Performance IQ and Verbal IQ scores with four secondary indices including: 1. Verbal Comprehension (information, similarities, and vocabulary). 2. Working Memory (arithmetic and digit span). 3. Perceptual Organization (picture completion, reasoning matrix, and block design). 4. Processing Speed (digit symbol-coding and symbol search).

In each subject, the following neurophysiological measures were also assessed: event-related potentials (P300), resting motor thresholds (rMT), active motor thresholds (aMT), and cortical silent periods (CSP). The examiner was blind to the degree of dementia and the experimental condition. All of the patients received memantine tablets 10 mg/day for at least 3 months before starting the study. All participants or their caregivers gave informed consent before participation in the investigation and after full explanation of the study protocol, which was approved by the Local Ethical Committee of Assiut University Hospital.

### Event-related potentials (P300)

Event-related potentials were elicited with an auditory discrimination task paradigm by presenting a series of binaural 2000 Hz (standard) vs. 1000 Hz (target) tones at 70 dB with a 10 ms rise/fall and 40 ms plateau time. Tones were presented at a rate of 1.1 per second, with target tones occurring randomly with 0.2 probabilities. The interstimulus interval was 3 s. The participant was required to distinguish between the two tones by responding to the target (pressing a button) and not responding to the standard tone. Evoked potentials were recorded from scalp electrodes placed at FZ (according to International 10–20 system) and were referred to linked ears A1/A2. Ground forehead electrode was applied at Fp1. Filter settings were 0.5 and 70 Hz, analysis time 1 s, sensitivity 20 μV and duration of stimulus 0.1 ms. Separate averages for target and non-target tones were obtained. Responses to 30 target and 120 non-target tones were obtained in each trial. Before recording, subjects were familiarized with the two tones. Automatically the number of errors was measured. The recordings of the responses were performed with a Nihon Kohden Machine model 9400 (Japan) with silver–silver chloride surface electrodes, applied at FZ. P300 latency was measured as the major positive peak after N200, within a range of 250–500 ms. P300 amplitude was measured peak to peak from the negative component just before P300, which represents N200, to the maximum positive peak P300.

### Electrophysiological investigations using TMS

Subjects sat in a comfortable chair. Electromyographic (EMG) recordings from the first dorsal interosseous muscle (FDI) of both hands were acquired with silver–silver chloride surface electrodes, using a muscle belly-tendon set-up, with a 3 cm diameter circular ground electrode placed on the wrist. A Nihon Kohden Machine model 9400 (Japan) was used to collect the signals. EMG parameters included a band pass of 20–1000 Hz and a recording time window of 200 ms. TMS was performed with a commercially available 90 mm figure-of-eight coil connected to Magstim super rapid magnetic stimulator (UK).

### Determination of motor thresholds

First, we determined the optimal scalp location in both hemispheres from which TMS evoked motor potentials of the greatest amplitude that could be evoked in the FDI. We used constant suprathreshold stimulus intensity and moved the figure-of-eight coil systematically in 1 cm steps to determine the scalp position from where TMS evoked motor potentials of maximum peak to peak amplitude in the target muscle. The coil was positioned tangentially to the scalp and oriented so that the induced electrical currents would flow approximately perpendicular to the central sulcus, at a 45 angle from the mid-sagittal line. Single pulse TMS was then delivered to the optimal location starting at suprathreshold intensity and decreasing in steps of 1% of the stimulator output. The EMG signals were monitored for 200 ms prior to stimulation. The rMT was defined as the minimal intensity required eliciting motor evoked potentials of 50 μV peak to peak amplitude in five out of ten consecutive trials.

The aMT was determined in the same way while subjects made a mild contraction of about 10% maximum. aMT was defined as the minimal intensity required to elicit an MEP larger than 200 μV in five of ten consecutive trials. Both the rMT and the aMT were expressed as a percentage of the magnetic stimulator output (maximum being equal to 100%). rMT and aMTs were measured in both hemispheres.

### Cortical silent period

The duration of the CSP was determined for left hemisphere during isometric voluntary contraction of the contralateral FDI. The participants were asked to perform a 50% maximum voluntary abduction of the index finger as judged by audio-visual feedback. Voluntary contraction started 10 s before TMS. Stimuli were delivered not closer than one every 15 s to avoid fatigue. Ten magnetic stimuli were applied at intensity 130% of rMT. The EMG traces were rectified and averaged. The length of the CSP (ms) was determined from the end of the motor evoked potential to the recurrence of at least 50% of EMG background activity.

All neurophysiological measures were made before the first and then repeated after the last treatment session.

### Group allocations: Three parallel groups

Anodal, cathodal, or sham with a ratio 1:1:1 were placed in serially numbered opaque closed envelopes. Each patient was given a serial number from a computer generated randomization table, and was placed in the appropriate group after opening the corresponding sealed envelope. The mean age (SD) of each group was 68.5 (7.2), 70.7 (5.4), 67.3 (5.9) years for anodal, cathodal, and sham group, respectively (*p* = 0.44) with male/female ratio. 6/5; 8/4; 5/6 for anodal, cathodal, and sham group, respectively (*p* = 0.59). There was no significant difference in the duration of dementia between groups (*p* = 0.81) with a mean duration (SD) of 3.0 (2.6), 2.9 (1.9), and 3.5 (1.7) years for anodal, cathodal, and sham group, respectively (Table 1).The anodal and cathodal groups received tDCS for 25 min at 2 mA daily for 10 consecutive days. The standardized stimulation localization was over the left DLPFC, at 6 cm anterior to the left primary motor cortex (M1) parallel to sagittal plane (George and Post, 2011; Li et al., 2013).

**Table 1:** **Demographic and clinical baseline data of the investigated groups**.

Demographic and clinical baseline data	Anodal group *N* = 11	Cathodal group *N* = 12	Sham group *N* = 11	*P* value
Sex male/female (19/15)	6/5	8/4	5/6	0.59
Age (years)	68.5 ± 7.2	70.7 ± 5.4	67.3 ± 5.9	0.44
Age of onset (years)	65.5 ± 7.8	68.3 ± 5.9	63.8 ± 5.9	0.27
Duration (years)	3.0 ± 2.6	2.9 ± 1.9	3.5 ± 1.7	0.81
MMSE score	18.4 ± 3.9	18.8 ± 2.9	16.9 ± 2.9	0.36
Dementia degree (mild/moderate) (15/19)	5/6	6/6	4/7	0.80
DM (yes/no) (8/26)	4/7	2/10	2/9	0.47
Hypertension (yes/no) (8/26)	1/10	4/8	3/8	0.36
IHD (yes/no) (2/32)	1/10	0/12	1/10	0.31
Neurological disease (yes/no)	3/8	1/11	0/11	0.50
Parkinsonism	2	1	0	
Senile tremors	1	0	0	

*MMSE score, mini-mental state examination; WAIS-III, Wichsler adult intelligent scale-third edition; DM, diabetes mellitus; IHD, ischemic heart disease*.

Direct current was delivered with saline soaked electrodes applied and secured onto the scalp (CX – 6650 Model TRCU – 04A Rolf Schneider Electronics, D-37130 Gleichen, Germany). The anodal electrode (24 cm^2^) was placed over the left DLPFC and the cathodal electrode (100 cm^2^ used as reference electrode) was fixed over the contralateral supraorbital region for anodal group. The electrode polarities were reversed for the cathodal stimulation group as the cathodal electrode (24 cm^2^) was placed over the left DLPFC and the anodal electrode (100 cm^2^ used as reference electrode) was fixed over the contralateral supraorbital region. A large reference electrode (100 cm^2^ compared to 24 cm^2^ for the functionally effective electrode) was used to reduce the possible effects of stimulation at that site by reducing the overall current density, as demonstrated by Nitsche et al. (2007).

For sham tDCS, the placement of the electrodes, current intensity, and ramp time was identical to atDCS stimulation group; however, the stimulation lasted only for 30 s. The investigator responsible for delivering tDCS had no contact with the patients. At the end of the therapy patients were asked whether they thought they had real tDCS or sham. Three of the real group and 2 of the sham group thought they had received real stimulation (chi squared test: *p* < 0.05), indicating that blinding was effective.

We followed up the patients clinically at the end of the 10th session (after measuring the cortical excitability) and then at 1 and 2 months after the end of the sessions. We also asked patients specifically whether they experienced any of the common side effects of tDCS, such as irritation under the electrodes, headache, and dizziness.

The change in MMSE was considered as primary outcome and changes in WAIS-III subtests and p300 were considered as secondary outcome. Cortical excitability and P300 were performed before the 1st tDCS session and repeated immediately after the end of 10th session for each patient. During the course of the study the patients did not receive any drug treatment except memantine 10 mg/day. All assessments were performed by a neurologist blind to the treatment group.

### Data analysis

All data were analyzed with the aid of the SPSS ver. 16 (http://www.spss.com). The results were expressed as mean ± SD. Since the distribution of the data did not differ statistically from normality, statistical analysis of the scores in each test was performed with a repeated measures analysis of variance (rmANOVA) with TIME (pre- and post-tenth session, and then at 1 and 2 months follow-up) as the within-subject factor, and TREATMENT CONDITION (anodal, cathodal, and sham tDCS) as the between subject measure. Effect of gender was also done along the course of treatment. Greenhouse–Geisser degree of freedom corrections were applied to correct for the non-sphericity of the data. *P* < 0.05 was considered significant for all statistical analysis. Spearman’s correlation was done between age and percent changes in MMSE (pre-post 3 months × 100/pre-stimulation).

## Results

All the patients tolerated tDCS well without any adverse effects except two patients under active stimulation recorded itching, headache, and dizziness that were disappear after few hours. Nineteen males and fifteen females withe no significant differences between groups as regards to sex distribution, with *P* = 0.59. There were no significant differences between the groups in baseline demographic and clinical data (age, onset, and duration of illness) or in the different clinical rating scales (MMSE and WAIS-III total scores) (see Table 1).

### Clinical scores

**Figure 2:**
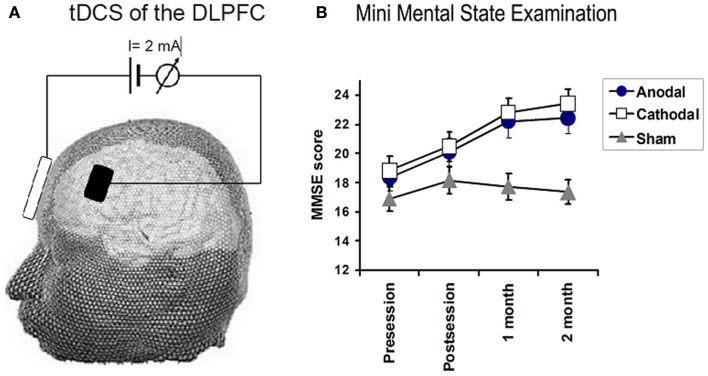
**(A)** Illustrates the technique used for transcranial DC stimulation. Direct current (2 mA) was applied between two, wet sponge-electrodes placed over the left dorsolateral prefrontal cortex (DLPFC) and the contralateral supraorbital region. TDCS polarity refers to the prefrontal electrode, which had a size of 24 cm^2^, whereas, the reference electrode had a size of 100 cm^2^. **(B)** Depicts the effects of tDCS on the mini-mental state examination (MMSE). Significant improvements in MMSE were observed in the anodal (*F* = 6.5, *P* = 0.01) and cathodal group (*F* = 13.8, *P* < 0.001), whereas no significant changes were observed in the sham group. A significant TREATMENT CONDITION × TIME interactions for MMSE (0.02).

Analysis of MMSE: there were main effects of TIME for each treatment group (*P* < 0.01 and 0.001 for anodal and cathodal group, respectively) while no such change was recorded in sham group (*F*_1.8_ = 1.0, *P* = 0.37). Two way rmANOVAs on the scores for the MMSE, with TREATMENT CONDITION_(Anodal, Cathodal, and Sham tDCS)_ and TIME_(baseline, post sessions, 1 month, and 2 months)_ as main factors revealed a significant TREATMENT CONDITION × TIME interactions for MMSE (*F*_3,50_ = 3.18, *P* = 0.029), mainly in orientation (*F*_3,9_ = 4.2, *P* = 0.005, registration (*F*_4,2_ = 4.7, *P* = 0.002), attention (*F*_4,0_ = 4.2, *P* = 0.004), and Naming objects (*F*_3,0_ = 6.8, *P* = 0.03) (see Figure 2).

Follow-up comparisons of each treatment group vs. sham revealed a significant TIME (pre, post, 1 month, and 2 months) × TREATMENT CONDITION interaction for the comparison of anodal vs. sham tDCS (*F*_2,52_ = 3.8, *P* = 0.04) and for cathodal vs. sham tDCS (*F*_2,52_ = 6.7, *P* = 0.005). There was no difference between cathodal and atDCS. We conclude that both forms of real tDCS improved MMSE score more than sham tDCS. We also conducted a number of exploratory analyses on subtests of the MMSE. Patients in the anodal group improved better than sham group in orientation, registration, attention, and in naming object *(F*_1.8_ = 3.5, *P* = 0.044, *F*_1.8_ = 5.8, *P* = 0.01, *F*_2.1_ = 7.3, *P* = 0.002, and *F*_1.3_ = 3.8, *P* = 0.05). No significant changes in the other subitems of MMSE.

Patients who received ctDCS improved significantly better than patients receiving sham along the course of the follow-up in the following tasks: orientation, registration, and attention (*F*_2,2_ = 10.7, *P* = 0.0001, *F*_2,2_ = 4.8, *P* = 0.01, *F*_1,8_ = 4.1, *P* = 0.02, respectively).

Analysis of WAIS-III (Full IQ, Performance and Verbal IQ) with TREATMENT CONDITION_(Anodal, Cathodal, and Sham tDCS)_ and TIME_(baseline, post sessions, 1 month, and 2 months)_ as main factors revealed a significant TREATMENT CONDITION × TIME interaction for WAIS performance scores (df = 3.229; *F* = 2.823; *P* = 0.04), but not for the verbal or total WAIS score (df = 4.254, *F* = 2.184, *P* = 0.076 and df = 4.717, *F* = 1.922, *P* = 0.105, respectively).

When each treatment group was tested separately against sham for effects on performance IQ, ANOVAs showed a significant TIME × TREATMENT CONDITION interaction when comparing cathodal vs. sham (*F* = 5.143, df = 2.123, *P* = 0.008) whereas this was not significant for atDCS (*F* = 1.149, df = 1.315, *P* = 0.253). However, there was no significant difference between anodal and cathodal effects. We conclude that ctDCS improved performance IQ more than sham tDCS.

Given the marginal significance of the overall ANOVA on verbal IQ (see above), we performed an exploratory analysis comparing each treatment group vs. sham. There was no significant TIME (pre, post, 1 month, and 2 months) × TREATMENT CONDITION interaction for anodal vs. sham tDCS (*F* = 1.06, df = 1.98, *P* = 0.354) or cathodal vs. sham tDCS (*F* = 2.762, df = 2.127, *P* = 0.071). However, it is interesting to note that, as with the performance IQ, there was a tendency for ctDCS to be slightly more effective than sham.

#### Effects of tDCS on the secondary indices of WAIS-III

**Figure 3:**
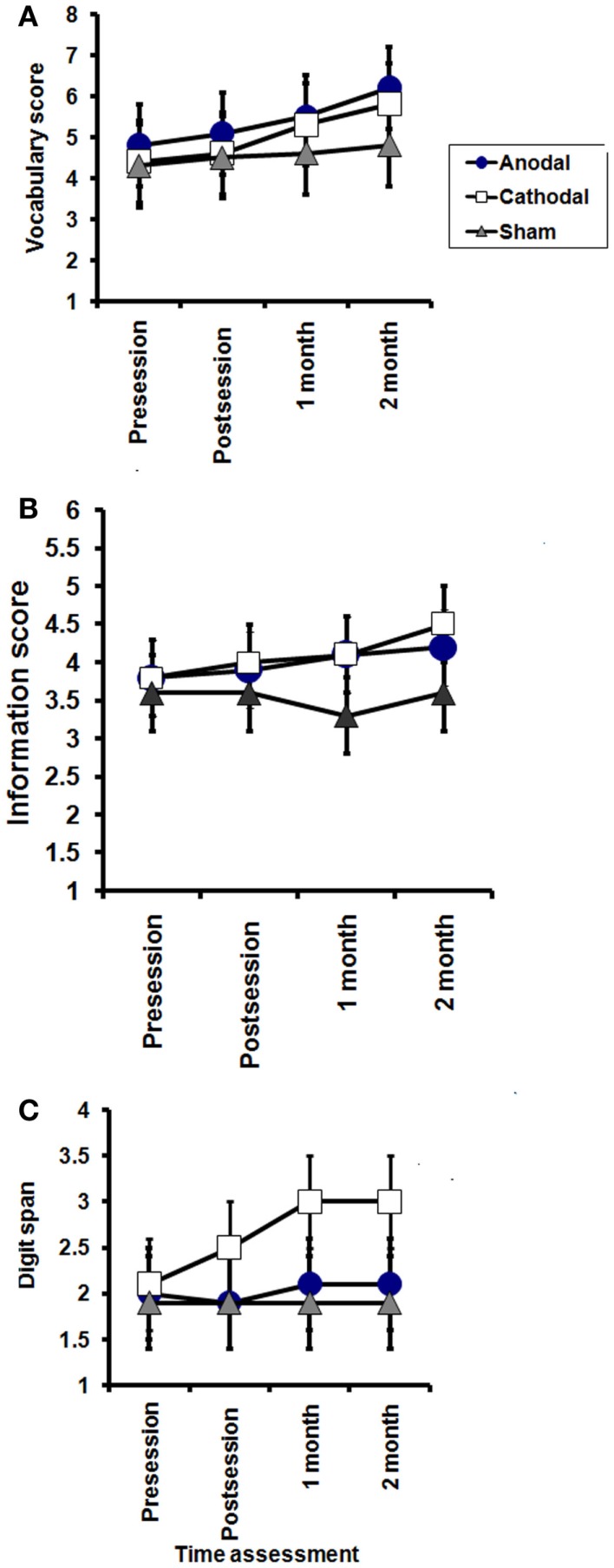
**Effects of tDCS on subscales of WAIS-III; vocabulary (A), information (B), and digit span (C)**. Vocabulary: significant effect of TIME in the anodal and cathodal groups (*P* = 0.03, and 0.007, respectively), while no significant effect of time on sham group (*P* = 0.1). Information: no significant effect of TIME (pre, post session, 1 month, and 2 months after end of sessions) in the anodal and sham groups (*P* = 0.4 and 0.6, respectively), while a significant improvement was found in the cathodal group (*P* = 0.01). Digit span; no significant effect of TIME (pre, post session, 1 month, and 2 months after end of sessions) in the anodal and sham groups (*P* = 0.8 and 0.1, respectively), while a significant improvement was found in the cathodal (*P* = 0.008), with no significant interaction between groups (time × groups) in any of these subscales.

For completeness, the data for the secondary indices are illustrated in Figure 3. Repeated measures two way ANOVA failed to reveal any significant GROUP × TIME interactions, suggesting that the effect of tDCS on WAIS is likely to be relatively small.

**Figure 4:**
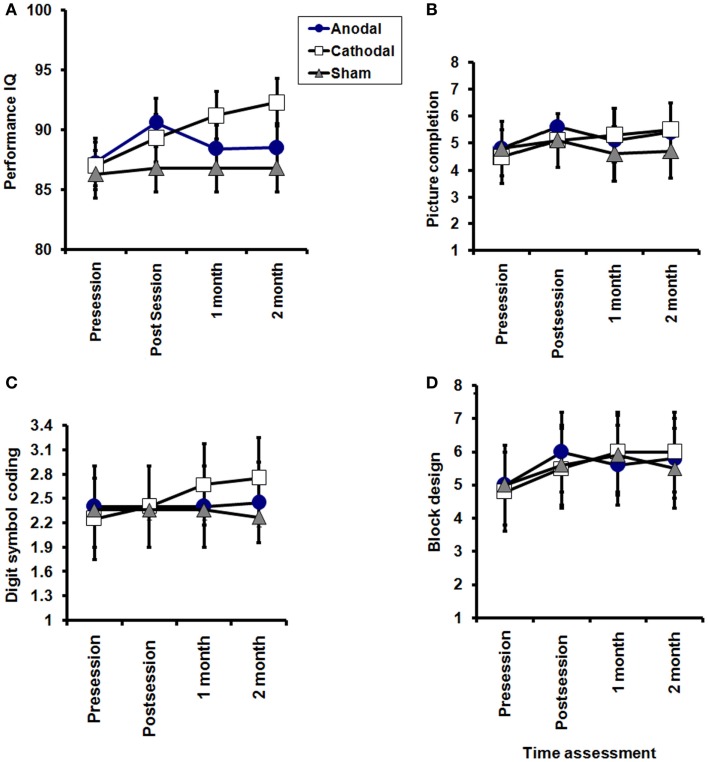
**Effects of tDCS on subscales of WAIS-III performance (A), picture completion (B), digit symbol-coding (C), and block design (D)**. A significant TREATMENT CONDITION × TIME interactions for WAIS performance (*P* = 0.04). Picture completion: significant effect of TIME (pre, post session, 1 month, and 2 months after end of sessions) in the anodal and cathodal groups group (*P* = 0.02 for each), while no significant improvement was sham group (*P* = 0.5). Digit symbol-coding: no significant effect of TIME (pre, post session, 1 month, and 2 months after end of sessions) in the anodal and sham groups (*P* = 0.6, 0.3, respectively), while a significant improvement was found in the cathodal group (*P* = 0.03). Block design: no significant effect of TIME (pre, post session, 1 month, and 2 months after end of sessions) in the anodal and sham groups (*P* = 0.2, and 0.1, respectively), while a significant improvement was found in the cathodal group (*P* = 0.0001) with no significant interaction between groups (time × groups) in any of the three subscales.

The results are depicted in Figures 3 and 4.

#### Effect of age and gender in relation to changes of different rating scales

No significant spearman’s correlation between age and percent changes in MMSE (pre-post 3 months × 100/pre-stimulation); *r* = 0.26 and *p* = 0.1790.

Two way ANOVA Time “pre, post, 1 month, and 3 months” × gender (male and female) show that; there is no effect of gender in relation to different rating scales along the course of treatment. For MMSE; df = 1.5 (48), *F* = 0.172, and *P* = 0.78. For verbal IQ df = 2.3 (65), *F* = 0.172, and *P* = 0.188 and for Performance IQ df = 1.6 (52), *F* = 1.1, and *P* = 0.31.

### Neurophysiological measures

A two factor rmANOVAs with TREATMENT CONDITION_(Anodal, Cathodal, and Sham tDCS)_ and TIME_(baseline, post sessions)_ revealed a significant TREATMENT CONDITION × TIME interaction for the P300 latency [df = 2 (31), *F* = 4.25, *P* = 0.023]. Follow-up pairwise comparisons showed significant effects of anodal and ctDCS over sham for the P300 latency [df = 1 (20), *F* = 5.9, and *P* = 0.02 for both], while there was no significant difference in P300 latency between atDCS and ctDCS (*F* = 0.7 and *P* = 0.79). Final one-way ANOVAs on the time course during each single intervention showed a significant effect of TIME for both anodal and ctDCS [df = 1 (10), *F* = 5.09, *P* = 0.041, and df = 1(11), *F* = 7.3, 0.03, respectively], while there was no significant change in the sham condition [df = 1 (10), *F* = 1.3, *P* = 0.21]. The P300 amplitude was unaffected by treatment [df = 2 (31), *F* = 0.05, *P* = 0.94].

**Figure 5:**
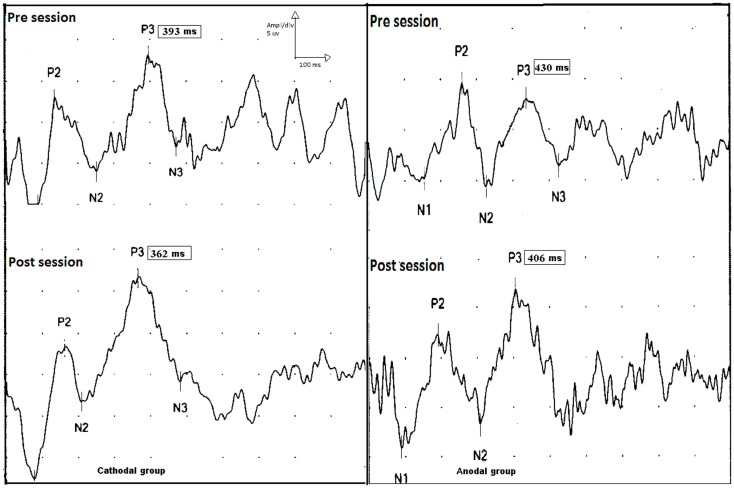
**P300 event-related potential, upper traces show p300 latencies and amplitudes before session (upper trace) in the cathodal (left side) and anodal stimulation (right side)**. Lower traces show the effect tDCS (post session) on the latencies of p300 on both groups. There is a shortening in p300 latencies post sessions in both groups (pre and post sessions for cathodal stimulation; 393 and 362 ms, and pre and post sessions 430 and 406 ms for anodal stimulation).

Concerning reaction time, there was a marginally significant TIME × TREATMENT GROUP interaction with df = 2(28), *F* = 2.7, *P* = 0.06. Exploratory one-way follow-ups of the effect of time in each group separately showed a each significant reduction of reaction time only after ctDCS [df = 1 (10), *F* = 9.4, and *P* = 0.01; see Table 2; Figure 5].

**Table 2:** **P300 latency, amplitude, and reaction time in pre and post sessions among the investigated groups**.

	Anodal group *N* = 11	Cathodal group *N* = 12	Sham group *N* = 11	*P* value time × groups (anodal vs. sham)	*P* value time × groups (cathodal vs. sham)	*P* value time × groups (anodal vs. cathodal)	*P* value time × groups (anodal/cathodal/sham)
**P300 latency (mean ± sd) in ms**							
Pre-session	379.8 ± 55.6	395.1 ± 64.2	350.5 ± 60.6	0.02	0.03	0.79	0.023
Post 10th session	336.5 ± 49.3	357.9 ± 62.5	371.2 ± 49.4	
*P* value *t*-test (pre–post sessions)	0.041*	0.034*	0.21	
**P300 amplitude (mean ± sd) in μv)**							
Pre-session	10.4 ± 6.5	8.2 ± 3.9	10.7 ±4	0.80	0.89	0.96	0.9
Post 10th session	9.9 ± 4.8	7.8 ± 3.1	9.7 ± 5.4	
*P* value *t*-test (pre–post sessions)	0.79	0.94	0.72	
**P300 reaction time (mean ± sd) in ms**							
Pre-session	569.9 ± 105.8	557.3 ± 71.8	569.7 ± 109.1	0.42	0.13	0.03	0.064
Post 10th session	585.4 ± 128.8	505 ± 72.8	559.5 ± 136.5	
*P* value *t*-test (pre–post sessions)	0.54	0.016	1				

*Values in the pre-session and post 10th session are expressed as mean ± SD*.

**P* values are obtained from two way ANOVAs with TREATMENT GROUP_(anodal, cathodal, and sham tDCS)_ as between subject factor and TIME_(pre-session and post 10th session)_ as within-subject factor*.

*P < 0.05.

Two way repeated measures ANOVAs revealed no significant TREATMENT CONDITION × TIME interactions for the active or rMTs of either left or right hemisphere. There were no significant changes in silent period (see Table 3).

**Table 3:** **Resting motor thresholds (rMT), active motor thresholds (aMT), and cortical silent period (CSP) from the left hemisphere in pre and post sessions among the investigated groups**.

	Anodal group *N* = 11	Cathodal group *N* = 12	Sham group *N* = 11	*P* value
**Resting motor threshold of left hemisphere (% of maximum output)**				
Pre-session	35.4 ± 7.2	37.8 ± 9.9	32.2 ± 7.1	0.454
Post 10th session	33.9 ± 7.8	36.8 ± 8.6	32.1 ± 7.5	
*P* value paired *t*-test (pre–post sessions)	0.035	0.2	0.86	
**Resting motor threshold of right hemisphere (% of maximum output)**				
Pre-sessions	33.18 ± 4.94	36.83 ± 9.97	32.82 ± 7.56	0.1
Post 10th session	31.91 ± 5.80	34.50 ± 7.53	32.09 ± 7.89	
*P* value paired *t*-test (pre–post sessions)	0.022	0.021	0.1	
**Active motor threshold of left hemisphere (% of maximum output)**				
Pre-session	26.1 ± 7.4	28.5 ± 7.6	27.6 ± 4.6	0.80
Post 10th session	25.4 ± 7.3	27.3 ± 6.3	27.2 ± 4.5	
*P* value paired *t*-test (pre–post sessions)	0.13	0.39	0.13	
**Active motor threshold of right hemisphere (% of maximum output)**				
Pre-sessions	24.64 ± 5.97	26.91 ± 6.61	25.64 ± 5.53	0.4
Post 10th session	22.82 ± 5.78	24.33 ± 5.33	24.73 ± 3.91	
*P* value paired *t*-test (pre–post sessions)	0.02	0.023	0.203	
**Cortical silent period (ms)**				
Pre-session	156.8 ± 38.3	147.3 ± 51.4	130.6 ± 37	0.87
Post 10th session	151.5 ± 37.4	144.8 ± 47.3	128.7 ± 40	
*P* value paired *t*-test (pre–post sessions)	0.26	0.21	0.17	

*Values in the pre-session and post 10th session are expressed as mean ± SD. *P* values are obtained from two way ANOVAs with TREATMENT GROUP_(anodal, cathodal, and sham tDCS)_ as between subject factor and TIME_(pre-session and post 10th session)_ as within-subject factor*.

## Discussion

Several recent studies have reported long-lasting, beneficial effects of high frequency rTMS over the DLPFC for patients with mild to moderate AD (Cotelli et al., 2006, 2008, 2011; Ahmed et al., 2012). The current randomized, double-blind, controlled study evaluated the effects stimulating left DLPFC with a different form of non-invasive brain stimulation, atDCS/ctDCS. We found that following 10 sessions of either atDCS or ctDCS followed by an additional 2 months of maintenance on memantine (10 mg), there was a significant improvement in the MMSE score in the active treatment groups, as compared with the sham group. The mean change was an increase of nearly 2 points immediately after the last treatment session with a further increase of 2 points at 1 and 2 months follow-up. In comparison, the mean change in the sham group was an increase of 1 point at the end of treatment, which declined by 0.8 and 0.4 point at 1 and 2 months, respectively.

The mechanism of the change is probably multifactorial. tDCS may increase the effectiveness of the cognitive reserve pool that is still operational in mild and moderate AD. This could involve effects of tDCS on a broad variety of neurotransmitters including acetylcholine and dopamine (Kuo et al., 2007; Monte-Silva et al., 2009), both of which are involved in cognitive function and behavior. A possible confound is that there is a practice effect on some of the clinical tests when repeated in short intervals. However, this is unlikely to be important since although the sham group improved at the first test point by a minor degree (1 point), the improvement faded on repeated testing at 1 and 2 months follow-up assessments.

Interestingly, both atDCS and ctDCS had similar effects. Although anodal and cathodal stimulation have opposite effects on motor cortex when applied with an intensity of 1 mA (Nitsche and Paulus, 2000; Liebetanz et al., 2002), in many therapeutic studies it is now standard to use 2 mA current, which was why we used it in the present work. For reasons that are so far unclear, at 2 mA ctDCS has the same excitatory effect on motor cortex as atDCS (Batsikadze et al., 2013; Wiethoff et al., 2014). Wiethoff et al. found that atDCS at 2 mA facilitated MEPs whereas there was no significant effect of 2 mA ctDCS. However, after two-step cluster analysis suggested that approximately 50% individuals had only a minor or no response to tDCS whereas the remainder had a facilitatory effect to both forms of stimulation. The reasons for this difference are unknown, and could involve explanations as diverse as the rates of calcium influx into pyramidal neurons or spread of effective current to connected brain regions (Batsikadze et al., 2013). Nevertheless if multiple mechanisms are involved in the response to ctDCS, then the less likely it is that any single mechanism will correlate with the overall after-effects (Wiethoff et al., 2014). Thus, our data on DLPFC are in line with those targeting the motor cortex (Nitsche et al., 2003; Batsikadze et al., 2013; Wiethoff et al., 2014). However, whether other cortical regions follow the same rule is unclear. For example, Monti et al. (2008) found that 2 mA ctDCS over left fronto-temporal areas significantly improved the accuracy of picture naming, whereas atDCS failed to induce any changes. Similarly, You et al. (2011) found that 2 mA ctDCS over right superior temporal areas induced significantly greater improvements in auditory verbal comprehension than atDCS or sham tDCS over left superior temporal areas.

Although stimulation was applied over DLPFC, it is difficult to predict the distribution of current that reaches the cortex (Neuling et al., 2012). It depends on factors including current density, modulation duration, electrode montage, electrode size, and orientation of the electric field in relation to the anatomical and geometrical features of the cortex. Indeed, widespread tDCS-induced changes in cortical activity have been demonstrated in previous neuroimaging studies (Keeser et al., 2011). However, since there were no effects on excitability of primary motor cortex in the present study, we think that there was at least partial focality of stimulation.

It has been argued that WAIS scores are not optimal for assessment of cognitive function of demented patients. Our data would be consistent with this since the effect of active tDCS on the WAIS was relatively minor, with the only significant effect being on the performance WAIS. Exploratory analysis of the subscale results suggested that there might be a larger effect of ctDCS than atDCS but these need further replication in a future studies.

The observed behavioral improvements were complemented by parallel changes in the P300 component of the ERP, which has been widely used to study age-related cognitive dysfunction, because it reflects attentional and memory processes. The P300 amplitude and latency are also correlated with the amount of attentional resources devoted to a given task (Wickens et al., 1983; Kramer and Strayer, 1988; Gonsalvez and Polich, 2002), and have been associated with superior memory performance (Fabiani et al., 1990; Johnson et al., 2004). P300 is also a measure of cognitive performance and decision making (Rohrbaugh et al., 1974; Pirtošek et al., 2009). In the present study, there was significant reduction of P300 latency for both forms of treatment groups as well as a reduced reaction time, only in the cathodal group. This result suggests the effect of tDCS on attentional and memory processes may be accompanied by measurable effects on cognitive event-related potentials. This result supports the Nakamura-Palacios et al. (2012) finding that tDCS of left DLPFC increase the mean P3 amplitude in different types of alcoholic patients.

Recent meta-analyses on the relation between the P300 component and AD showed that there is an increase in P300 latency in elderly patients with AD compared with subjects without the disease (Pedroso et al., 2012). Moreover, it has recently been shown that P300 latencies correlate significantly with performance on the MMSE and the Consortium to Establish a Registery for Alzheimer’s disease (CERAD), thus it has been argued that the P300 could be used as a biological marker to indicate impaired neuropsychological functions in AD patients (Lee et al., 2013). Our finding that 2 mA ctDCS/atDCS of the DLPFC resulted not only in cognitive improvements but also in a significant reduction of the P300 latency in AD patients is therefore of some clinical relevance. It can be assumed that altered local cortical excitability in one part of the responsible network influences the whole neural network associated with cognitive functions beyond the site of stimulation leading to comparable electrophysiological effects.

In conclusion, the results of this preliminary study demonstrate that both atDCS and ctDCS of the left DLPFC at 2 mA can not only improve cognitive functions, but also reduce the P300 latency in AD patients. These findings extend the results of previous studies and open the way for further exploration of the use of brain stimulation in the rehabilitation of AD. Moreover, as far as we know, this is the first study using tDCS in an Arabic country (Egypt) demonstrating that with limited research facilities tDCS can be used as a simple and effective way to modulate cortical excitability in neuropsychiatric disorders.

Limitations of the study include the small sample size and possible effects of concurrent depression as well as the use MMSE and WAIS-III are global measurements and not site-specific for the DLPFC. In addition, all patients received the NMDA receptor antagonist memantine, which can potentially affect induction of plasticity with tDCS. Further studies on unmedicated patients using more specific psychometric tests for assessment of executive function of DLPFC would help resolve this.

## Author Contributions

Eman M. Khedr contributed to study concept and design, acquisition of data, draft and revision of the report, statistical analyses, and interpretation of data. Nageh F. El Gamal, Noha Abo El-Fetoh, Hosam Khalifa, Anwer M. Ali, Mostafa Noaman, and Ahmed Abd El-Baki contributed to acquisition of data, statistical analyses, and interpretation of data. Elham M. Ahmed contributed to study acquisition, analysis of data. Ahmed A. Karim contributed to study revision of the report, analyses, and interpretation of data.

## Conflict of Interest Statement

The authors declare that the research was conducted in the absence of any commercial or financial relationships that could be construed as a potential conflict of interest.
